# Cassowary casques act as thermal windows

**DOI:** 10.1038/s41598-019-38780-8

**Published:** 2019-02-13

**Authors:** Danielle L. Eastick, Glenn J. Tattersall, Simon J. Watson, John A. Lesku, Kylie A. Robert

**Affiliations:** 1 Department of Ecology, Environment and Evolution, La Trobe University, Melbourne, Victoria, 3086 Australia; 2 Department of Biological Sciences, Brock University, St. Catharines, Ontario, L2S 3A1 Canada

## Abstract

Many ideas have been put forward for the adaptive value of the cassowary casque; and yet, its purpose remains speculative. Homeothermic animals elevate body temperature through metabolic heat production. Heat gain must be offset by heat loss to maintain internal temperatures within a range for optimal performance. Living in a tropical climate, cassowaries, being large bodied, dark feathered birds, are under thermal pressure to offload heat. We tested the original hypothesis that the casque acts as a thermal window. With infrared thermographic analyses of living cassowaries over an expansive range of ambient temperatures, we provide evidence that the casque acts as a thermal radiator, offloading heat at high temperatures and restricting heat loss at low temperatures. Interestingly, at intermediate temperatures, the casque appears thermally heterogeneous, with the posterior of the casque heating up before the front half. These findings might have implications for the function of similar structures in avian and non-avian dinosaurs.

## Introduction

The cassowary is a large, flightless bird that bears a prominent helmet (or casque). The function of the casque has attracted considerable curiosity and speculation for nearly 200 years^1^, yet its purpose remains unclear^2^. Early descriptions of the casque referred to it as a horny structure^3,4^, a horn-covered bony growth^5^ or hoof-like material^6^, giving rise to the most widely recognised idea that the casque is used as a protective structure for moving at high speed in dense vegetation^5,7,8^ and during fights with other animals^5^. Others have suggested that the casque is a secondary sexual characteristic^9^, although this hypothesis might not reflect the reason for its origin since both males and females are casque-bearing. Alternatively, the casque has been proposed to act as a resonance chamber to amplify the cassowary’s low frequency boom^10^; however, anatomical data argues against this idea^11^. One report^12^, of a single cassowary offered a possible thermal function, but the casque was outside the focus of the study, and the animal was studied only at ambient temperatures (T_a_) less than 30 °C.

Homeothermic animals maintain a largely stable internal body temperature that is often different from T_a_ through the metabolic production of heat^13,14^. Many animals have evolved morphological structures, or adapted existing body regions, known as ‘thermal windows’, for heat exchange^13,15–20^. A pre-requisite that enables a structure to exchange heat with the environment is that it must be highly vascularised and uninsulated^13,21,22^ to enable some of the warmth in blood to be dissipated. Vessels in these body parts are superficial, facilitating heat exchange before the blood is recirculated towards the core. It is crucial that blood flow is adjustable to these regions^13,17^. At low T_a_, vessel walls constrict, limiting blood flow to the area and enabling the warm blood to flow within the body. At warmer T_a_, vessels dilate, which facilitates the cooling of blood before returning to the deep interior of the body.

The cassowary casque meets the characteristics of thermal windows: uninsulated and vascularised. Casques are keratinized, overlying a body crown and network of trabeculae surrounded by dorsoventrally aligned canals containing blood vessels making up an extensive vascular network^2,6,9^. Moreover, the cassowary faces a thermal challenge owing to its large size (up to 160 cm height; with females (60 kg) heavier than males (30 kg)), dark plumage and tropical distribution in Oceania^23–25^. For these collective reasons, it is possible that the distinctive helmet-like structure upon the cassowary’s head acts as a thermal radiator to remove excess heat. We provide evidence that the casque serves (at least in part) as a thermal window. This finding reinforces the possibility that Mesozoic dinosaurs with similar structures^26–28^ may have also used such appendages to cope with tropical environments.

## Results

### The cassowary has thermal windows

**Figure 1:**
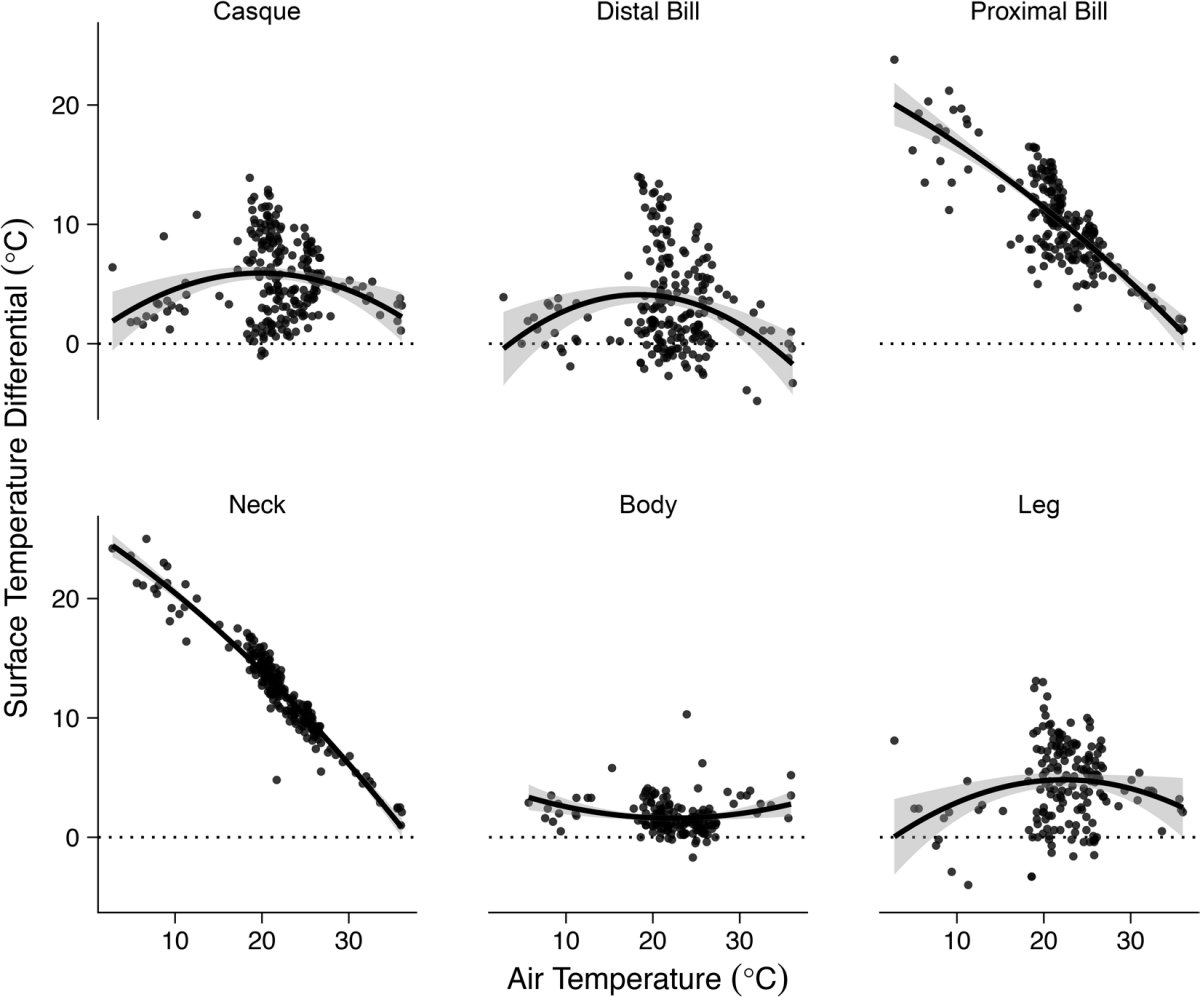
Surface temperature differentials of cassowary body regions over a range of air temperatures (5–36 °C). Points reflect raw data; the line is the model fit; grey shading denotes 95% confidence interval. The eye is not presented as it followed a similar pattern to the proximal bill and neck.

To better understand whether cassowary’s use their casque for temperature homeostasis, we conducted infrared thermographic analyses of 20 live cassowaries over an expansive range of ambient temperatures (T_a_). Consistent with this prediction, we found that the casque displayed evidence of reactive vasomotion across different heat loads. Indeed, the casque, distal end of the bill, and legs all showed a capacity for thermal adjustment (Fig. 1). These regions showed relatively large differentials between appendage surface temperature and T_a_ at intermediate T_a_, and smaller thermal contrasts at either end of T_a_ extremes. Conversely, the body was minimally affected by changes in T_a_, owing to insulation from the feathers. The proximal bill and neck displayed a linear negative relationship with increasing T_a_ suggesting no active regulation of blood flow to these surfaces. This is not unexpected since at a T_a_ of 10 °C, the differential is ~20 °C because the surface temperatures of both regions are ~30 °C. Similarly, at a T_a_ of 30 °C, the differential declines to ~5 °C because the neck and proximal bill are still close to 30 °C. In this way, these regions are not making large thermal adjustments.

**Figure 2:**
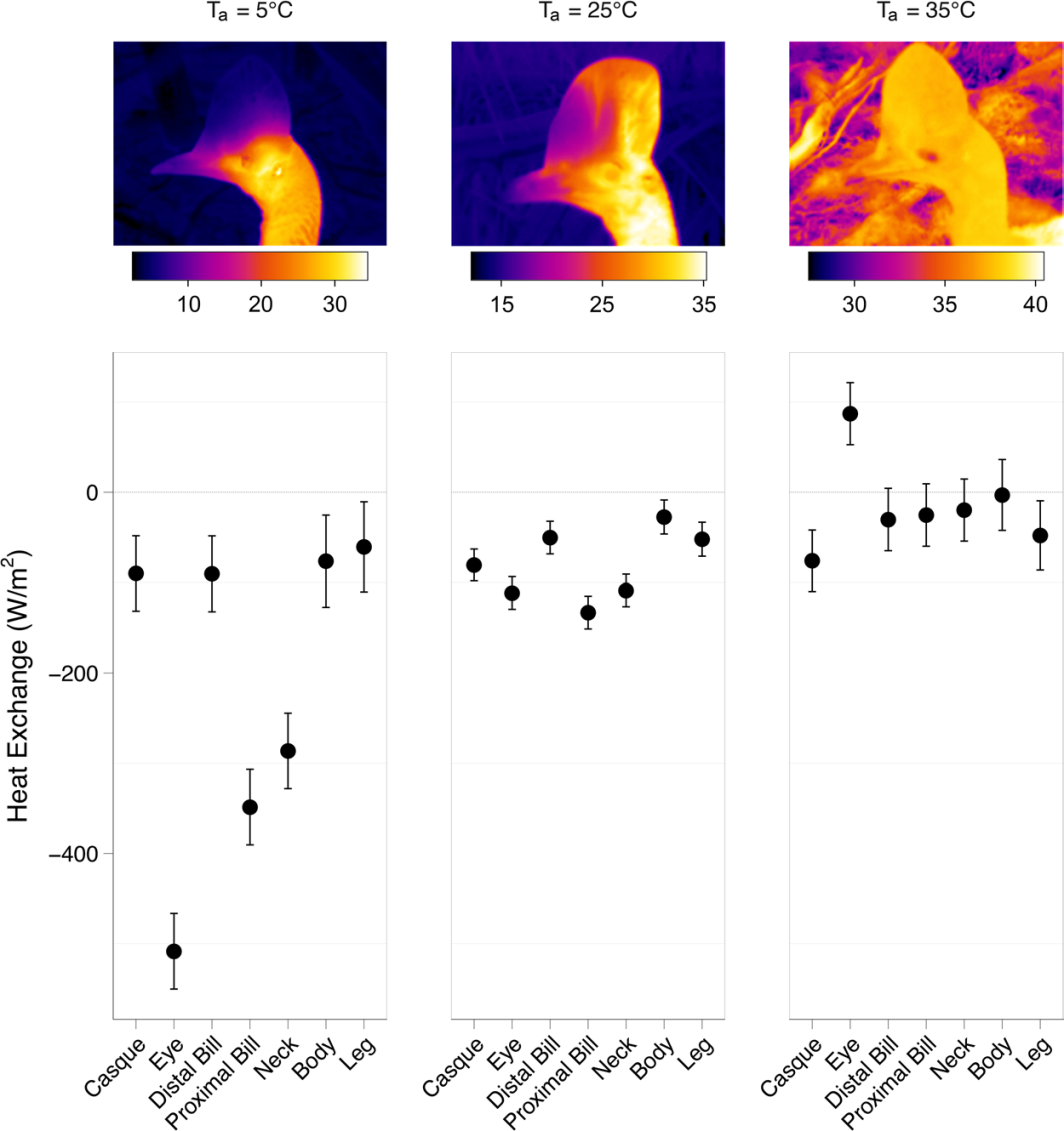
The effects of ambient temperature (T_a_) on heat exchange of seven body appendages. Data are presented as mean with 95% confidence interval. Negative values denote heat loss, whereas positive means reflect heat gain. *Top*, representative thermal images are presented showing thermal profiles of the casque in different T_a_. The cassowary in the 5 and 35 °C photographs is the same bird.

The capacity for thermal adjustment translated into propensity for heat exchange, although body surfaces varied significantly in their ability to exchange heat with the environment (Fig. 2, Table S1, χ^2^ = 741.70, d.f. = 6, P < 0.001). Notably, the surfaces of the cassowary most similar to core body temperature (i.e., the uninsulated eye, neck and proximal bill) were thermal windows at low T_a_, losing heat to the cold environment. The ability of a body region to serve as a thermal window depended on T_a_ (χ^2^ = 420.30, d.f. = 6, P < 0.001). For instance, the body appendages from which the most heat was lost at low T_a_ also lost less heat (per m^2^) at the highest temperatures.

### The casque is the most important thermal window at high heat loads

**Figure 3:**
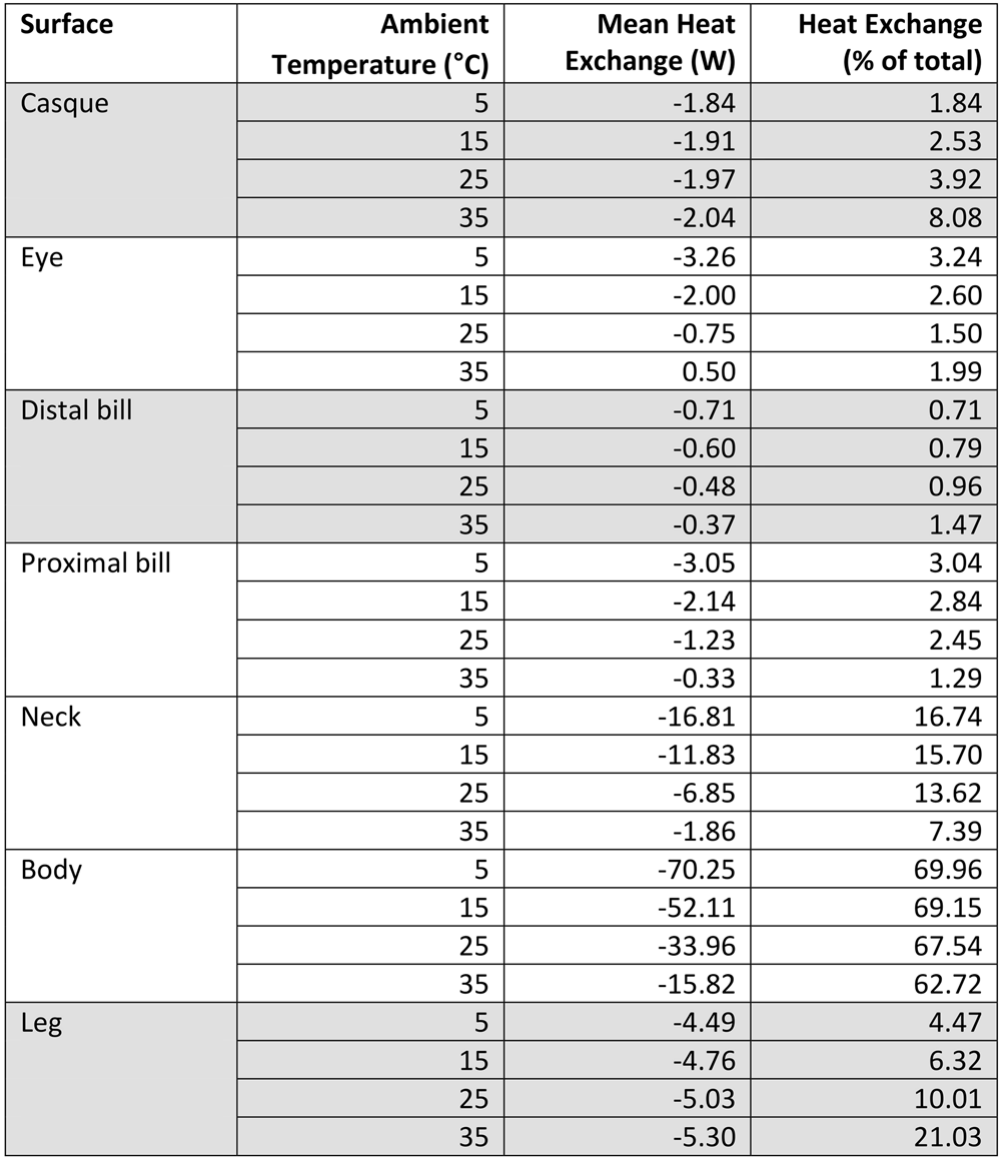
Measures of heat exchange across seven body surfaces occurring under four ambient temperatures. A linear mixed model fit provided a predicted mean heat exchange (Q_total_, *W*). Heat exchange is represented as a percentage of heat exchanged by each appendage (W). Negative values denote heat loss; positive values reflect heat gain. Grey highlight denotes body regions that increased heat loss (as a % of total) with increasing T_a_.

**Figure 4:**
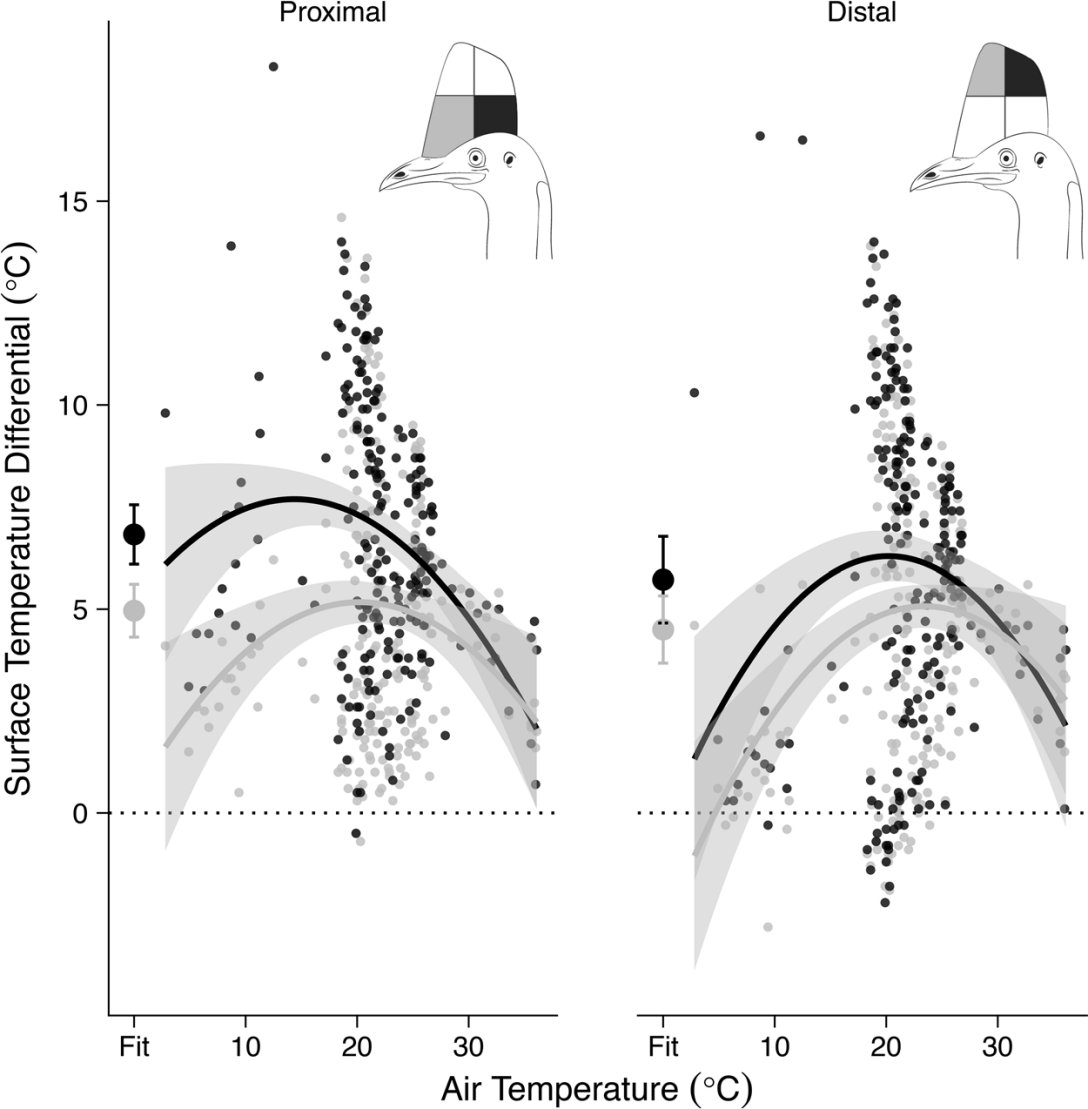
Thermal profiles of the four quadrants of the cassowary casque under a range of air temperatures. Inset: Cassowary illustrations show the anterior (grey) and posterior (black) regions of the casque for proximal and distal quadrants. Grey shading around each line reflects 95% confidence interval (CI); the fit points are the model fit with 95% CI. Cassowary head illustration courtesy of Breanna Eastick (used with permission).

Importantly, at the highest T_a_, the casque dissipated the greatest amount of heat (per m^2^) to the environment (Fig. 2). The distal bill and legs likewise increased the amount of heat lost to the environment at increasing T_a_, at least when represented as a function of total heat exchange (Fig. 3). Moreover, the casque offloaded more heat to the environment than the eye, bill (distal and proximal) and neck at high T_a_. Indeed, heat loss across the casque accounted for 8% of all heat exchange, a disproportionately high value given the relatively small size of the casque. In this way, the casque served as a substantial thermal window when the animal was under high thermal loads. This process is best illustrated by thermal images of the casque over cold, moderate and hot T_a_ (Fig. 2). At low and high T_a_, the surface temperature of the casque approximates T_a_, likely owing to vasoconstriction and vasodilation, respectively, of casque vasculature. The thermal images also revealed a mosaic of thermal adjustments at moderate T_a_, wherein the posterior (back) part of the casque tended to heat up before the anterior half. We explored this pattern by dividing the casque into quadrants to examine changes in temperature profiles across the casque. The four casque regions (χ^2^ = 95.42, d.f. = 3, P < 0.001; Fig. 4; Table S2) displayed significantly different heat dissipation patterns. At moderate T_a_, the proximal posterior quadrant of the casque was hotter than the proximal anterior region (see also the thermal image in Fig. 2 at 25 °C), a pattern not reflected in the distal portions of the casque (χ^2^ = 92.69, d.f. = 6, P < 0.001).

## Discussion

The function of the casque has remained enigmatic for nearly 200 years^1,29^. Our results confirm our hypothesis that the cassowary can regulate heat exchange via the casque. Indeed, cassowaries use their casque much in the same way as some other birds use their beaks for heat exchange^13,20,30^. Here, cassowaries used the casque as a thermal window when ambient temperature (T_a_) was high, but restricting heat loss when T_a_ was low. It is noteworthy that the cassowaries were observed dunking their heads into water at high T_a_ (D.L.E. pers. obs.), perhaps to further increase cooling of the blood in the casque. Interestingly, at intermediate T_a_ (25 °C), the temperature profile of the casque was heterogenous with the posterior of the casque heating up before the front half. Separate vessels may be supplying the anterior and posterior regions, enabling asynchronous dilation and constriction of vessels, as seen in the toco toucan’s (*Rhamphastos toco*) bill^13^. Computed tomographic scanning reveals anterior-posterior differences in the amount of trabeculae in the cassowary casque, with dense trabeculae at the front that becomes increasingly diffuse towards the back^11^. Nonetheless, additional imaging would be useful to reconstruct the arterial and venous pathways of the casque vasculature, as has been used to (virtually) dissect avian vascular anatomy in other species^31^.

Body surfaces that track T_a_ are not considered to be actively involved in heat exchange, such as the feathers. The large proportion of total heat exchange by the body is simply a function of its large surface area. The eye, neck, and proximal bill likewise exchange some heat with the environment, but these regions are not specialised for that purpose. Instead, they maintain a consistently high surface temperature independent of environmental variation. Conversely, other body regions vary greatly in temperature over a range of T_a_, and can therefore be considered specialised for heat exchange^12^. Specifically, the casque, distal bill and legs were thermal windows.

Allen’s rule hypothesizes that animals in cooler environments are expected to have smaller appendages, relative to whole-body size^32^. Appendages tend to lose the most heat, thus enlarged appendages will have increased overall heat dissipation, while conservation of heat is associated with reduced appendage size^32–34^. This holds true for bill size in many birds, which are often larger in birds from warmer environments than those from cooler environments^20^. Casques and casque-like structures are also present on birds inhabiting warmer habitats, for example hornbills (Bucerotiformes: Bucerotidae)^30^, helmeted guineafowl (*Numida meleagris*)^35^ and the maleo (*Macrocephalon maleo*)^36^. Interestingly, pterosaurs and some dinosaurs, including members of the Ornithischia and Saurischia had similar cranial adornments^26–28^. While the function of these structures is still poorly understood, our results suggest these dinosaurian ornaments may have also served a thermoregulatory role. Hence, findings about the function of the casque in extant species could complement current and future findings in fossilised species with similar structures.

## Methods

### Collection of data

Live, adult southern cassowaries (*Casuarius casuarius*; n = 20) were photographed with a hand-held thermal imager (Testo 875i, Testo Ag, Lenzkirch, Germany) in zoological parks ranging from Victoria in southern Australia (outside of their natural range) to Queensland in northern Australia, between April 2014 and September 2015 (austral autumn and spring, respectively). These parks were chosen to encompass a wide thermal range without the need to restrain birds within temperature controlled facilities, which would be logistically unlikely owing to their protected status and dangerous disposition. Meteorological conditions were recorded using a wireless weather station (Vantage Pro 2 Plus, Davis Instruments Australia, Pty. Ltd., Kilsyth, Australia) situated within 10 metres of the cassowary’s enclosure.

Thermal images were taken from a distance of 0.5–2 metres. The camera had a double lens which produced a thermal image and a digital image at a resolution of 160 × 120 pixels. Images were collected between 0800–1700 h covering a wide range of ambient temperatures (T_*a*_), 5–36 °C. Temperature data from the images were measured using IRSoft 3.1 (Testo Ag, Lenzkirch, Germany). Average surface temperature of the casque, eye, bill (distal and proximal), neck, body (torso) and ‘legs’ (tarsometatarsus only as the tibiotarsus is covered with feathers) were calculated, assuming an emissivity of 0.96. Mean surface temperatures were measured using either a line (neck, legs, body), point (eye), circle (distal bill; around nostrils, and proximal bill; anterior to eye) or by tracing the area (casque). The casque was further divided into four regions: the distal anterior, proximal anterior, distal posterior and proximal posterior. A total of 2,487 images were used.

The La Trobe University Animal Ethics Committee approved the study (AEC14-11) and all methods were carried out in accordance with the relevant guidelines and regulations.

### Calculation of heat exchange

Heat exchange (*q*; W/m^2^) estimates were calculated according to previously published studies^37^, and using the Thermimage package (v. 3.1) in R^38,39^. The estimates of heat exchange (loss = negative, gain = positive) were calculated as the sum of the convective and radiative heat exchange from a particular body surface, incorporating local measurements of ambient temperature, solar radiation, wind speed, and relative humidity. Operative temperature experienced by the cassowaries was not estimated due to an inability to safely measure the precise local microenvironment near the bird at the time of image capture. To visually inspect differences among surfaces, we plotted estimates of heat exchange at three values of ambient temperature spanning the ranges of measured ambient temperatures. Total heat exchange (Q) was estimated by multiplying heat exchange by area values (m^2^) obtained from adult specimens found in museums. To assess the proportional role of the casque in heat loss and gain, we fit linear mixed models of Q_casque_ as a function of Q_total_, and used the slope estimates as an indicator of the proportion of heat exchanged by the appendage.

### Statistical analyses

To quantify the influence of ambient temperature on regional differences in casque surface temperature, we fit linear mixed effects models^40^, incorporating the casque surface of interest (broken into 4 quadrants based on proximal-distal and posterior-anterior designations) as a fixed effect and animal identity as a random effect, with image identity nested within animal identity. Following the estimation of body surface specific heat fluxes, we fitted linear mixed effects models of the area specific heat exchanges (W/m^2^), using body surface of interest (eye, neck, proximal bill, distal bill, mean casque, leg, and body), ambient temperature, and time of day as fixed effects, animal identity as a random effect, with image identity nested within animal identity. Surface and ambient temperature interactions were also assessed in the above model. In all cases, residuals were verified for normality and homoscedasticity. Finally, to estimate the relative contributions of the casque to heat exchange, we compared the model estimates from total heat exchange for the casque to the total body heat exchange estimates, as well as to published estimates of basal heat production in similarly sized emus^41^. We used model fits (±95% confidence interval) as measures of support and to summarise results. P-values were obtained using likelihood ratio tests (Type II Wald’s chi-square tests) using the *car* package in R^42^.

## Supplementary information

Supplemental Information

## Data Availability

The datasets generated during and/or analyzed during the current study are availble from the corresponding author on reasonable request.
