# Supplementary material for: Cassowary casques act as thermal windows
Source: Sci Rep. 2019 Feb 13;9:1966. doi: 10.1038/s41598-019-38780-8 (PMC6374359; doi:10.1038/s41598-019-38780-8)
Supplement: Supplementary file 1 — Supplemental Information [file 41598_2019_38780_MOESM1_ESM.docx]

**SUPPLEMENTAL INFORMATION**

**Cassowary casques act as thermal windows**

Danielle L. Eastick^1*^, Glenn J. Tattersall^2^, Simon J. Watson^1^, John A. Lesku^1^ and

Kylie A. Robert^1^

^1^Department of Ecology, Environment and Evolution, La Trobe University, Melbourne, Victoria, 3086, Australia

^2^Department of Biological Sciences, Brock University, St. Catharines, Ontario, L2S 3A1, Canada

*Author for correspondence:

Danielle Eastick

La Trobe University ⏐ Kingsbury Drive (BS1) ⏐ Bundoora 3086 ⏐ Australia

T: +61 4 8854 6625 ⏐ E: d.eastick@latrobe.edu.au

This document includes:

Supplementary Statistical Tables

**Table S1.** Analysis of Deviance (type II Wald *χ*^2^ test) across seven appendages: casque, eye, distal bill, proximal bill, neck, body and leg.

| Parameters | *χ^2^* | d.f. | P-value |
| --- | --- | --- | --- |
| Surface | 741.70 | 6 | < 0.001 |
| AmbTemp | 53.60 | 1 | < 0.001 |
| Surface * AmbTemp | 420.30 | 6 | < 0.001 |

Model fit: Q_total_ ~ Surface * AmbTemp + (1 | ID/Image)

**Table S2.** Analysis of Deviance (type II Wald *χ*^2^ test) for each quadrant of the casque: posterior proximal and distal, and anterior proximal and distal.

| Parameters | *χ^2^* | d.f. | P-value |
| --- | --- | --- | --- |
| poly(AmbTemp, 2) | 6.11 | 2 | 0.0472 |
| Surface | 95.42 | 3 | < 0.001 |
| Hour | 5.57 | 1 | 0.0183 |
| poly(AmbTemp, 2) * Surface | 92.69 | 6 | < 0.001 |

Model fit: ∆T ~ poly(AmbTemp, 2) * Surface + Hour + (1 + Surface | ID) + (1 | ID/Image)
